# Factors That Can Promote the Green Entrepreneurial Intention of College Students: A Fuzzy Set Qualitative Comparative Analysis

**DOI:** 10.3389/fpsyg.2021.776886

**Published:** 2022-03-08

**Authors:** Xinhai Cai, Shahid Hussain, Yuying Zhang

**Affiliations:** ^1^School of Computer and Control Engineering, Yantai University, Yantai, China; ^2^Department of Management Science, Karakoram International University, Gilgit, Pakistan; ^3^School of Public Administration, Dongbei University of Finance and Economics, Dalian, China

**Keywords:** green entrepreneurial intention, entrepreneurship education, college students, fsQCA, sustainable development

## Abstract

Green entrepreneurship has a huge role in solving environmental degradation and social problems. As today’s youth are tomorrow’s entrepreneurs, enhancing their green entrepreneurial intention will contribute to the sustainable development of economy in the future. The existing literature has examined the green entrepreneurial intention of college students based on self-efficacy, entrepreneurial creativity, entrepreneurship education, financial support, sustainable development values, and other influencing factors. However, these studies focus on net effect of factors on the results of college students’ green entrepreneurial intention, ignoring the combination of multiple factors, and the relationship between different configurations of factors and the results of college students’ green entrepreneurial intention. Therefore, this study aimed to understand and analyze the influence of the complex relationship between multiple antecedents (entrepreneurial creativity, future self-continuity, green cognition, entrepreneurship culture, entrepreneurship education, and financial support) on college students’ green entrepreneurial intention from a comprehensive perspective. Based on 207 questionnaires collected from colleges in the coastal cities of China, this study used the fuzzy set qualitative comparative analysis method for data analysis. The results showed three dominant paths of great significance to college students’ high green entrepreneurial intention: entrepreneurial creativity—green cognition—dominant path; entrepreneurial creativity—financial support—dominant path; and the future self-continuity—entrepreneurship culture—entrepreneurship education—dominant path. The results of this study can help college educators to plan courses on innovative thinking and green entrepreneurship to improve college students’ entrepreneurial creativity and green cognitive ability.

## Introduction

Studies on green economy have revealed that it can effectively solve environmental deterioration and that green entrepreneurship is an important part of this concept (Soomro et al., 2020). Green entrepreneurship is the entrepreneurial process of identifying and creating “unrealized” products and services that promote sustainable development of the environment (Cohen and Winn, 2007; Dean and McMullen, 2007). Green entrepreneurship helps to reduce the damage to natural resources, solve environmental problems (Silajdzic et al., 2015), and provide new employment opportunities (Dean and McMullen, 2007), which further help in promoting sustainable social and economic development. Green entrepreneurs can potentially instill green entrepreneurship among college students and help them practice it (Soomro et al., 2020; Alvarez-Risco et al., 2021).

College students’ green entrepreneurial intentions are influenced by many factors (Bruton et al., 2010; Zhou and Verburg, 2020). Schumpeter’s innovation theory states that innovation is a new combination of products, production methods, markets, and materials, in which creativity is the core content of innovative activity and the key factor of green entrepreneurial intention (Jiang et al., 2020). As green entrepreneurship involves the identification of entrepreneurial opportunities and sustainable development aligned with the environment, it is necessary to further develop green technology and green entrepreneurial thinking. Thus, entrepreneurs may have to face more challenges in the process of green entrepreneurship. Future self-continuity is also an important factor that affects entrepreneurial choice. Individuals with higher future self-continuity can accept more uncertainty during the entrepreneurial process (Zeng and Ouyang, 2020). Additionally, the individual sustainable development values (Kuckertz and Wagner, 2010; Ploum et al., 2018; Vuorio et al., 2018; Sher et al., 2020; Thelken and de Jong, 2020; Agu et al., 2021; Yasir et al., 2021) and entrepreneurship education for sustainable development (Lans et al., 2014; Wijnker et al., 2015; Soomro et al., 2020; Agu et al., 2021) are the most important factors that influence the green entrepreneurial intention of college students. The positive sustainable development values of individuals and the sustainable development entrepreneurship education programs set up by colleges may encourage college students to adopt green entrepreneurship. In addition, diversified financial support, such as low interest loans and campus entrepreneurship funds, can further promote green entrepreneurship among them (Butkouskaya et al., 2020). External environmental support (Fichter and Tiemann, 2018; Vuorio et al., 2018; Yi, 2020), personality (Yan et al., 2018; Wu et al., 2019), self-efficacy (Sher et al., 2020; Alvarez-Risco et al., 2021), and entrepreneurial enthusiasm (Tehseen and Haider, 2021) have a significant impact on college students’ green entrepreneurial intention. The existing literature that explores college students’ green entrepreneurship is based on internal factors (e.g., personality and individual characteristics of entrepreneurs) or external factors (e.g., entrepreneurial environment, policy support, entrepreneurship education). From the perspective of research methods, these studies mainly use structural equation models (Soomro et al., 2020; Thelken and de Jong, 2020; Alvarez-Risco et al., 2021; Yasir et al., 2021) and regression analysis (Vuorio et al., 2018; Butkouskaya et al., 2020), and focus more on the marginal “net effect” of independent variables on dependent variables. Therefore, they cannot explain the complex causal relationships between multiple factors.

The advantage of the fuzzy set qualitative comparative analysis (fsQCA) method is that it can make up for the methodological limitations in the existing research and reveal the influence of the complex relationship between multiple antecedent conditions on the outcomes. However, in the face of many factors that affect college students’ green entrepreneurial intention, the question is “how to choose conditional variables?” Among the various methods to select conditional variables, the most widely accepted one is to summarize conditional variables based on the same or different research theoretical perspectives. In addition, the selection of the number of conditional variables should be reasonable. Too many conditional variables would easily lead to the “individualized” interpretation of the case. Usually, the number of conditional variables is approximately 4–7. This study selected the conditional variables based on Bandura’s theory of reciprocal determinism (Bandura, 1986). The theory holds that the interaction of behavior, personal subject factors, and environmental factors affect people’s behavior. College students’ green entrepreneurial intention can be explained through Bandura’s reciprocal determinism. Based on this theory, college students are the behavioral subject of entrepreneurial activities. Based upon the interaction of personal and environmental factors may affect their green entrepreneurial intention. They may be expected to perform entrepreneurial activities in the future owing to their personal ability. Entrepreneurial environment can have a subtle impact on individual entrepreneurial behavior and a good environment can stimulate entrepreneurial intention. The existing literature categorizes the factors that influence college students’ green entrepreneurial intention as internal and external factors. The internal factors refer to personal factors, which include personal entrepreneurial creativity and future self-continuity that affect college students’ green entrepreneurial intention. External factors refer to the formal and informal institutional framework in the entrepreneurial process, which mainly comprises three dimensions: the cognitive pillar, the regulatory pillar, and the normal pillar (Busenitz et al., 2000). The cognitive pillar refers to the knowledge regarding entrepreneurial activities; regulatory pillar refers to the laws and regulations that promote entrepreneurial activities; and normal pillar refers to whether entrepreneurial environment, entrepreneurial culture, and beliefs have a positive orientation to entrepreneurial activities, which will impact entrepreneurial decision-making. Based on the institutional research framework of Xie et al. (2021), this study identified four influencing factors: green cognition, entrepreneurship culture, entrepreneurship education, and financial support.

In this context, starting from the entrepreneur’s individual characteristics and institutional theory framework, this study used the fsQCA method to explore the causal mechanism of collaborative configuration of antecedents (influencing factors) on college students’ green entrepreneurial intention. The key problems to be solved in this study are as follows:

A. What are the core and marginal factors that affect the green entrepreneurial intention of college students?
B. Which path can promote the green entrepreneurial intention of college students?
C. What is the relationship between the different paths?

The contribution of this study is manifold. First, it enriches the application of the research method of college students’ green entrepreneurial intention. Previous studies mainly discussed green entrepreneurial intention based on structural equation method. In this study, fsQCA method was used to replace independent variables with conditional configuration and set relationship instead of correlation. The combination of quantitative and qualitative analyses makes up for the shortcomings of earlier research. Moreover, it enriches the theoretical scope of future self-continuity in college students’ green entrepreneurial intention. Current research on future self-continuity focuses mainly on the effects of saving and consumption behavior, academic achievement, intertemporal choice, and health research. Only one study shows that individuals with high future self-continuity are more likely to adhere to entrepreneurship in the future (Zeng and Ouyang, 2020). This study elaborates on the factors influencing college students’ green entrepreneurial intention and expands the theoretical application of future self-continuity in college students’ green entrepreneurial intentions. The fsQCA approach also enhances the research perspective of college students’ green entrepreneurial intention. From the perspective of overall cognition and configuration, it breaks the “net effect” thinking that the existing literature is limited to factors for the results. This study explores the complex impact of the relationship between entrepreneurial creativity, future self-continuity, green cognition, entrepreneurship culture, entrepreneurship education, and financial support on the outcome.

Later, we review the relevant literature and research framework of individual entrepreneur characteristics and institutional theory framework, followed by our proposed research methods and processes. Then we present the results. Lastly, we analyze the implications of the study, identify its shortcomings, and discuss and summarize the results of this study.

## Literature Review and Research Framework

### Literature Review

#### Entrepreneurial Creativity and Green Entrepreneurial Intention

Entrepreneurial creativity refers to the ability of individuals to produce and implement innovative ideas regarding products and services in early or mature enterprises to establish new enterprises, plan the development direction of enterprises, and produce new services and products (Amabile, 1997). It is original and novel and requires entrepreneurs to find a new thinking system from existing achievements, to better explore and find the connection between divergent aspects, and creatively find new business opportunities (Zhan et al., 2020). Entrepreneurial creativity is not only the key driving force of entrepreneurial intention but also the core element for enterprises to improve their competitive strength (Olufunso, 2010). As a special group, green entrepreneurs should maintain vigilance to protect the environment, pay attention to the environmental development problems that other entrepreneurs do not realize, use innovative thinking to find and identify business opportunities, open up the entrepreneurial market, and employ flexible creative thinking to solve environmental problems. Studies have shown that college students’ entrepreneurial creativity has a positive impact on entrepreneurial intention (Zampetakis, 2008). Compared to students with average creativity, those with higher creativity are better able to break the inherent thinking of problems. Thus, they are more likely to show positive entrepreneurial spirit (Liang et al., 2019), higher green entrepreneurial willingness (Jiang et al., 2020), and the possibility of implementing green entrepreneurial behavior. Therefore, entrepreneurial creativity has an important impact on college students’ green entrepreneurial intention.

#### Future Self-Continuity and Green Entrepreneurial Intention

The related connotation of self-continuity was mentioned by the philosopher Parfit (1971), who pointed out that an individual’s past experience will affect the past self and eventually become a part of self-identity; the individual’s view of the future self will also affect the present self. Therefore, as an individual identifies the past, present, and future selves as one self in the time dimension, it becomes a continuous self. Ersner-Hershfield et al. (2009) put forward the connotation of future self-continuity and believed that its essence refers to the closeness of the individual’s perception of the present and future selves, by focusing on a person’s feelings and views of the present and future selves. Hershfield (2011) summarized the characteristics of future self-continuity as vividness, similarity, and positivity. The psychological and behavioral aspects of entrepreneurs are impacted by the consistency of their future self-continuity. The higher the degree of psychological connection between the future and the present selves, the more the individual is able to adhere to the present decision (Ersner-Hershfield et al., 2009) in the future choice. The individual can also come into a positive emotional state to make efforts for future decisions. In addition, some researchers have shown that future self-continuity is an entrepreneurial trait of start-ups. Individuals with high future self-continuity are likely to accept uncertain entrepreneurial environment and adhere to entrepreneurship in the future environment than others (Zeng and Ouyang, 2020).

#### Green Cognition and Green Entrepreneurial Intention

Entrepreneurial cognition refers to the cognitive structure of individuals in the process of creating new enterprises and identifying and evaluating market opportunities (Smith et al., 2009). Green cognition refers to the process of individual understanding and understanding of the green concept and psychological experience of undertaking green problems through the integration of green knowledge and skills accumulated by individuals (Jiang et al., 2020). The existing literature shows that green cognition is positively correlated with green entrepreneurial intention. The stronger the entrepreneur’s green cognition, the more likely the person will have green entrepreneurial intention and exhibit various green entrepreneurial behaviors. Entrepreneurs with high green awareness are better than ordinary entrepreneurs in terms of environmental awareness. Green entrepreneurs pay more attention to whether entrepreneurial behavior and decision-making have a negative impact on the environment. Therefore, when they start a business, they look for and identify green business opportunities. However, entrepreneurs with good green cognition can identify potential green entrepreneurial interests and green ideas and implement and solve the problems of a green economy (Jiang et al., 2020). Thus, college students’ cognition of green entrepreneurship, including their understanding of green concepts, cognition of environmental problems, and the ability to identify green entrepreneurial opportunities, may affect their green entrepreneurial intention.

#### Entrepreneurship Culture and Green Entrepreneurial Intention

Entrepreneurship culture refers to beliefs, attitudes, and values related to entrepreneurial activities in a specific region or country (Linan et al., 2020). Different modes of entrepreneurship reflect the variations in cultural modes in many countries and the entrepreneurship culture advocated by the regions where the entrepreneurs live (Capelleras et al., 2019). Social and cultural norms affect individuals’ entrepreneurial intention. Some researchers point out that if a country or region encourages entrepreneurship and shows cultural respect for the identity of entrepreneurs, and if individuals can actively feel the entrepreneurial spirit of struggle, innovation, and risk-taking, then they are more willing to try entrepreneurship, which can promote individual entrepreneurial intention (Spencer and Gomez, 2004). Green entrepreneurial culture with social support can affect entrepreneurs’ green entrepreneurial spirit and entrepreneurial quality (Alwakid et al., 2020). By actively developing green entrepreneurial culture, beliefs, and norms, a country and society can better convey the entrepreneurial development direction to entrepreneurs, which directly or indirectly affects the choice of individual entrepreneurial direction (Diaz-Casero et al., 2012). The promotion of green entrepreneurial culture is guided by social norms and policies. Therefore, the green entrepreneurial intention of college students is promoted, when the country, colleges, and universities actively advocate a green entrepreneurship culture.

#### Entrepreneurship Education and Green Entrepreneurial Intention

Entrepreneurship education refers to the national education system that provides entrepreneurship-related education for students, which allows them to identify entrepreneurial opportunities and possess entrepreneurial skills (Kuratko, 2005). The level of entrepreneurship education is positively correlated with entrepreneurial intention (Mark, 2012). Thus, it is an important determinant of students’ entrepreneurial intentions (Agu et al., 2021). Green entrepreneurship education refers to the cultivation of entrepreneurs with environmental and economic values, thus emphasizing the concept and practice orientation of green entrepreneurship. College students with basic knowledge of green entrepreneurship are more willing to participate in green entrepreneurship activities (Yi, 2020). It may be because they are gradually able to learn green knowledge and entrepreneurial skills in the diversified green entrepreneurship education organized by the school. Green entrepreneurship education can further stimulate the construction of college students’ cognition of green entrepreneurship and encourage them to deal better with the possible problems faced in the process of green entrepreneurship in the future, so as to enhance their green entrepreneurial intention. Therefore, green entrepreneurship education is also an important factor that affects college students’ green entrepreneurship.

#### Financial Support and Green Entrepreneurial Intention

Financial support mainly refers to subsidies, loan discounts, tax incentives, and other financial support for actively carrying out green entrepreneurship activities. Owing to the special orientation of “environmental values,” green entrepreneurship needs more financial support than other forms of entrepreneurship (Welter, 2011). Financial support is inevitable for the normal operation of the project (Xu et al., 2020). In particular, green entrepreneurship requires more green technology and financial support is the basic guarantee for green entrepreneurs. When college students realize that there is a lack of financial support or funds, they rather delay starting or wanting to start a business (Braun and Sieger, 2021). However, the implementation of financial policies by the state, government, colleges, and universities, make them more willing to accept financial support and avail of more entrepreneurial resources and opportunities. The fundamental function of financial support is to provide effective material guarantees to reduce the uncertainties caused by technology and market risks at the initial stage or in the process of entrepreneurship, to enhance their confidence and enthusiasm in entrepreneurship (Pergelova and Angulo-Ruiz, 2014; Xu et al., 2020). Therefore, venture capital support is more conducive to stimulating the practice of green entrepreneurship among college students and has an important impact on their green entrepreneurial intention.

### Research Framework

**FIGURE 1:**
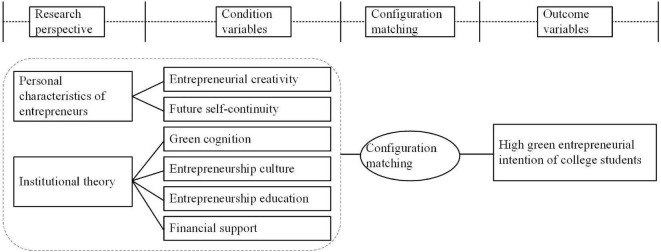
Research framework.

In summary, the single motivational factor of college students’ green entrepreneurship provides the basis for the condition selection in this study. However, no study has explored the influence of complex configuration among various factors on college students’ green entrepreneurial intention. In this context, based on the fsQCA method, this study constructs an explanatory framework and research model from the perspective of entrepreneurial creativity, future self-continuity, green cognition, entrepreneurship culture, entrepreneurship education, and financial support, and examines how these six factors affect college students’ green entrepreneurship, as shown in Figure 1.

## Materials and Methods

### Method

This study used fsQCA to explore the impact of entrepreneurs’ individual characteristics and institutional theory framework on college students’ green entrepreneurial intention from the perspective of configuration. The advantage of fsQCA is that it combines the advantages of qualitative and quantitative analyses (Ragin, 2006). Its basic principle is to analyze the influence of different combinations of multiple antecedent variables on the results based on Boolean operation to explain the complex causal mechanism that affects the results. It is suitable for small samples (less than 10 or 15), medium samples (10 or 15–50), and large samples (more than 100). The ideal number of condition variables were approximately 4–7. Too less condition variables lead to the result being too simple and meaningless; too many condition variables lead to complex results, which cannot effectively be further refined and discussed in detail. Fiss (2011) pointed out that for a specific research result, its influencing factors are multifaceted, rather than the unique contribution of a single condition. Therefore, fsQCA can analyze the factors influencing college students’ green entrepreneurial intentions in more detail. It is helpful to explore several effective guidance programs to activate college students’ high green entrepreneurial intention.

### Producers and Participants

We distributed questionnaires to different types of colleges in the coastal cities of China. Coastal cities were chosen owing to their rapid economic development accompanied by ecological and environmental problems. Therefore, these cities give great importance to green entrepreneurship for economic growth and green development (Shang and Liu, 2021). We randomly selected seven different types of colleges in seven coastal cities. About 30 students were sampled from each college. Stratified sampling was adopted, and approximately 7 to 8 students were sampled from each grade. To ensure the effectiveness of questionnaire collection, seven college teachers were entrusted with distributing questionnaires. The completed questionnaires were collected after the stipulated time. Before filling the questionnaire, the participants were assured of anonymity and confidentiality.

Each questionnaire was divided into two parts: demographic measure (e.g., gender, school, and grade), condition variable (entrepreneurial creativity, future self-continuity, green cognition, entrepreneurship culture, entrepreneurship education, and financial support) and outcome variable (green entrepreneurial intention). The five demographic questions were as follows: (1) Which is/are your gender? (2) Are you an only child? (3) What grade are you in? (4) Do you have any practical experience in entrepreneurship in school? and (5) Do your parents (or other immediate family members) have any experience in starting a business? A total of 220 questionnaires were collected, of which 207 were valid, accounting for 94.09% response rate. Among 207 samples, 97 (46.86%) college students were male and 110 (53.14%) were female; 63 (30.43%) were single child and 144 (69.57%) non-single child; there were 20 students in grade one (9.66%), 22 students in grade two (10.63%), 102 students in grade three (49.28%), and 63 students in grade four (30.43%); 50 (24.15%) college students had entrepreneurial practice experience, 157 (75.85%) had no entrepreneurial practice experience; and 62 (29.95%) college students’ parents had entrepreneurial experience, 145 (70.05%) college students’ parents had no entrepreneurial experience.

### Measurement Instruments

#### Entrepreneurial Creativity

Chia and Liang (2016) developed a scale for entrepreneurial creativity, which included 12 items. Each item was rated on a 5-point Likert scale with the options (1 = “very much disagree” to 5 = “very much agree”). In this study, the scale showed good reliability (Cronbach’s alpha = 0.930). The items of the measurement scale were revised include (“I can plan innovative entrepreneurial activities;” “I can plan entrepreneurial activities with my characteristics;” “I can plan stimulating entrepreneurial activities;” “Entrepreneurial activities that I plan are ingenious;” “Entrepreneurial activities that I plan are unique;” “Entrepreneurial activities that I plan are to guide the market;” “I understand customers’ needs;” “I adapt practices flexibly to the changes;” “I consider preferences in the consumer market;” “Entrepreneurial activities that I plan are to meet customers’ goals;” “Entrepreneurial activities that I plan can be adapted to different situations;” “Entrepreneurial activities that I plan are recognized in the consumer market”).

#### Future Self-Continuity

Future self-continuity was measured on a 10 items scale adapted from Sokol and Serper (2020). It is divided into three structures: the similarity questions (Question 1–4; e.g., “How similar are you now to what you will be like 10 years from now?” “How similar are your beliefs now to what they will be like 10 years from now?” “How similar is your personality now to what it will be like 10 years from now?” “How similar are your values now to what they will be like 10 years from now?”) are answered on a 5 - point Likert scale (1 = “completely different” to 5 = “exactly the same”), the vividness questions (Questions 5–7; “How vividly can you imagine what you will be like in 10 years from now?” “How vividly can you imagine what you will look like in 10 years from now?” “How vividly can you imagine what your family relationships will be like in 10 years from now?”), and positive affect questions (Questions 8–10; “Do you like what you will be like 10 years from now?” “Do you like what your personality will probably be like 10 years from now?” “Do you like what your actions will probably be like 10 years from now?”), and the 5-point Likert scale is used to answer (1 = “not at all” to 5 = “perfectly”). The average score of each future self-continuity questionnaire item was used to obtain the total score of the future self-continuity questionnaire (ranging from 1 to 5). The higher the score, the higher the future self-continuity level. This scale has been widely used in previous studies and has good reliability, with a Cronbach’s alpha of 0.921.

#### Green Cognition

Jiang et al. (2020) compiled the scale of green cognition, which has six items (“I can recognize new venture opportunities in environmental protection industries;” “I frequently identify ideas that can be converted into new products or services in environmental protection industries;” “I generally lack green ideas that may materialize into profitable enterprises;” “I frequently identify opportunities to start up new businesses in environmental protection industries;” “I enjoy thinking about new ways of doing green businesses;” “I thought of many ideas for new green activities in the past month”). The third question is the reverse. The responses for the questions were based on a 5-point Likert scale (1 = “very disagree” to 5 = “very agree”) and with a Cronbach’s alpha of 0.828.

#### Entrepreneurship Culture

The entrepreneurship culture scale has been widely used in research (Diaz-Casero et al., 2012), with a total of 5 items (“In my country/region, the national culture is highly supportive of individual success achieved through own personal efforts;” “In my country/region, the national culture emphasizes self-sufficiency, autonomy, and personal initiative;” “In my country/region, the national culture encourages entrepreneurial risk-taking;” “In my country/region, the national culture encourages creativity and innovativeness;” “In my country/region, the national culture emphasizes the responsibility that the individual (rather than the collective) has in managing his or her own life”). The score of each item ranges from 1 (very disagree) to 5 (very agree), and has shown good reliability with a Cronbach’s alpha of 0.930.

#### Entrepreneurship Education

The Walter and Block (2016) scale was used in entrepreneurship education, with a total of 4 items (“My school education helped me develop my sense of initiative—a sort of entrepreneurial attitude;” “My school education helped me to better understand the role of entrepreneurs in society;” “My school education made me interested to become an entrepreneur;” “My school education gave me skills and know-how that enable me to run a business”). The responses were based on a 5-point Likert scale which ranged from 1 (very disagree) to 5 (very agree). In this study, the Cronbach’s alpha was 0.726.

#### Financial Support

The financial support scale was compiled by Wu and Mao (2020), and there were five items in total. The fourth question was related to the competition intensity of local financial institutions, which had nothing to do with this study; so, it was deleted. Finally, four questions were included in this study (“There are alternative finance sources for student entrepreneurs;” “Low-interest loans offered by banks are accessible;” “Start-up funds offered by the school/government are accessible;” “A variety of loan guarantee options are available”). The score of each item ranged from 1 (very disagree) to 5 (very agree). In this study, the Cronbach’s alpha was 0.873.

#### Green Entrepreneurial Intention

The green entrepreneurial intention scale is a 6-item scale compiled by Wang et al. (2016). The six questions are (“I will do anything to become a green entrepreneur;” “My professional goal is to become a green entrepreneur;” “I will make every effort to establish and operate my own green business;” “I am seriously considering starting a green business;” “I am determined to become a professional green business manager;” “I am committed to developing my green business into a high-growth enterprise”). Each item is scored on a 5-point Likert scale and used to answer the questions (1 = “very disagree” to 5 = “very agree”). In this study, the green entrepreneurial intention scale had good reliability (Cronbach’s alpha = 0.778). All these items are shown in Table 1.

**TABLE 1:** The related items of condition variables and outcome variable.

Research levels	Variables	Item	References
Outcome variable	Green entrepreneurial intention	1. I will do anything to become a green entrepreneur. 2. My professional goal is to become a green entrepreneur. 3. I will make every effort to establish and operate my own green business. 4. I am seriously considering starting a green business. 5. I am determined to become a professional green business manager. 6. I am committed to developing my green business into a high-growth enterprise.	Wang et al., 2016
Condition variable	Entrepreneurial creativity	1. I can plan innovative entrepreneurial activities. 2. I can plan entrepreneurial activities with my characteristics. 3. I can plan stimulating entrepreneurial activities. 4. Entrepreneurial activities that I plan are ingenious. 5. Entrepreneurial activities that I plan are unique. 6. Entrepreneurial activities that I plan are to guide the market. 7. I understand customers’ needs. 8. I adapt practices flexibly to the changes. 9. I consider preferences in the consumer market. 10. Entrepreneurial activities that I plan are to meet customers’ goals. 11. Entrepreneurial activities that I plan can be adapted to different situations. 12. Entrepreneurial activities that I plan are recognized in the consumer market.	Chia and Liang, 2016
	Future self-continuity	1. How similar are you now to what you will be like 10 years from now? 2. How similar are your beliefs now to what they will be like 10 years from now? 3. How similar is your personality now to what it will be like 10 years from now? 4. How similar are your values now to what they will be like 10 years from now? 5. How vividly can you imagine what you will be like in 10 years from now? 6. How vividly can you imagine what you will look like in 10 years from now? 7. How vividly can you imagine what your family relationships will be like in 10 years from now? 8. Do you like what you will be like 10 years from now? 9. Do you like what your personality will probably be like 10 years from now? 10. Do you like what your actions will probably be like 10 years from now?	Sokol and Serper, 2020
	Green cognition	1. I can recognize new venture opportunities in environmental protection industries. 2. I frequently identify ideas that can be converted into new products or services in environmental protection industries. 3. I generally lack green ideas that may materialize into profitable enterprises. (reverse) 4. I frequently identify opportunities to start up new businesses in environmental protection industries. 5. I enjoy thinking about new ways of doing green businesses. 6. I thought of many ideas for new green activities in the past month.	Jiang et al., 2020
	Entrepreneurship culture	1. In my country/region, the national culture is highly supportive of individual success achieved through own personal efforts. 2. In my country/region, the national culture emphasizes self-sufficiency, autonomy, and personal initiative. 3. In my country/region, the national culture encourages entrepreneurial risk-taking. 4. In my country/region, the national culture encourages creativity and innovativeness. 5. In my country/region, the national culture emphasizes the responsibility that the individual (rather than the collective) has in managing his or her own life.	Diaz-Casero et al., 2012
	Entrepreneurship education	1. My school education helped me develop my sense of initiative—a sort of entrepreneurial attitude. 2. My school education helped me to better understand the role of entrepreneurs in society. 3. My school education made me interested to become an entrepreneur. 4. My school education gave me skills and know-how that enable me to run a business.	Walter and Block, 2016
	Financial support	1. There are alternative finance sources for student entrepreneurs. 2. Low-interest loans offered by banks are accessible. 3. Start-up funds offered by the school/government are accessible. 4. A variety of loan guarantee options are available.	Wu and Mao, 2020

### Data Processing

#### Calibration of Variables

The calibration of variables refers to the process of collective membership of grouping the condition variables (entrepreneurial creativity, future self-continuity, green cognition, entrepreneurship culture, entrepreneurship education, financial support) and the outcome variable (green entrepreneurial intention), which transforms the original data of the condition and outcome variables into a fuzzy set membership score of 0–1. Three critical values need to be set: the fully in point, crossover point, and fully out point (Ragin, 2008). In this study, the data were calibrated according to the standard proposed by Fiss (2011): 75% (fully in), 50% (crossover point), and 25% (fully out). First, the percentile of the data was calculated using SPSS, and the thresholds of the fully in point, the crossover point, and the fully out point were set to the 75th, 50th, and 25th percentiles of each variable, respectively. The calibration anchors for each variable are listed in Table 2. Furthermore, the calibration function in fs/QCA3.0, was used to calibrate each variable, with each variable set after calibration belonging to 0∼1.

**TABLE 2:** Each variable calibration anchor and sample descriptive statistics.

Research levels	Variables	Abbreviation	Anchor point			Measure descriptives			
			Fully in	Crossover point	Fully out	*M*	*SD*	Max	Min
Condition variable	Entrepreneurial creativity	ECR	3.8330	3.1670	2.9170	3.3136	0.7356	5.000	1.000
	Future self-continuity	FSC	4.0000	3.2000	2.9000	3.3643	0.7261	5.000	1.550
	Green cognition	GC	3.8330	3.500	3.0000	3.5250	0.6650	5.000	2.000
	Entrepreneurship culture	ECU	4.6000	3.8000	3.0000	3.7401	0.7936	5.000	2.000
	Entrepreneurship education	EE	4.0000	3.2500	2.7500	3.3019	0.7152	5.000	1.500
	Financial support	FS	4.0000	3.0000	2.2500	3.0797	1.1346	5.000	1.000
Outcome variable	Green entrepreneurial intention	GEI	3.6670	3.1670	2.6670	3.2061	0.6046	5.000	1.8330

*M, mean; SD, standard deviation. Data in the table were calculated using SPSS 23.0.*

#### Necessity Analysis

If the necessary condition is included in the truth table analysis, it may be eliminated by parsimonious solution. The consistency threshold of the necessity analysis is 0.9. If the condition variable exceeds 0.9, it indicates that the condition variable constitutes a necessary condition. When an outcome occurs, a condition always exists (when the condition is missing, the outcome cannot occur). Then, the explanatory power of this condition variable on the outcome variable is stronger than that of the other condition variables. Table 3 shows the necessity analysis results for college students’ high green entrepreneurial intention. Because the consistency of each condition is not higher than 0.9, there is no need for separate conditions for high green entrepreneurial intention.

**TABLE 3:** The necessity of conditions for high green entrepreneurial intention.

Condition tested	Consistency	Coverage
ECR	0.787418	0.788191
∼ECR	0.331534	0.321072
FSC	0.697223	0.702393
∼FSC	0.421238	0.405441
GC	0.796643	0.800651
∼GC	0.329866	0.318216
ECU	0.791638	0.834126
∼ECU	0.381097	0.352040
EE	0.764942	0.776991
∼EE	0.383256	0.366014
FS	0.568947	0.573507
∼FS	0.556580	0.535403

#### Truth Table Analysis

The fuzzy set truth table algorithm of the fsQCA software is used to select the outcome and condition variables of the model, and the truth table is generated automatically. Furthermore, the consistency threshold and case threshold of the variables must be set. Variable consistency refers to the probability that the combination of condition variables has a decisive explanatory power on the outcome variable. The higher the threshold, the higher the explanatory probability. A consistency threshold of 0.8 indicates a good and robust subset relation (Fiss, 2011; Bell et al., 2014; Campbell et al., 2016). Case coverage refers to the number of cases that can be covered by a specific condition variable, a combination of variables, or the number of acceptable cases. The larger the value, the more cases in which the condition variable can lead to key results. In general, the case threshold is set to 1 (Ragin, 2008; Hernandez-Perlines et al., 2016). In addition, we need to review the proportional reduction in inconsistency (PRI) to reduce the contradictory configurations. If it was less than 0.7, the outcome variable was set to 0. Finally, based on fs/QCA3.0, three types of solutions were obtained: complex, intermediate, and parsimonious. The intermediate solution reflects the easy counterfactuals (consistent with theory or substantive knowledge), while the parsimonious solution reflects difficult counterfactuals (inconsistent with theory or substantive knowledge). The existence of both intermediate and parsimonious solutions can determine the core conditions.

## Results

### Configuration Analysis

As shown in Table 4, seven configurations (GEI1a, GEI1b, GEI1c, GEI2, GEI3a, GEI3b, and GEI3c) produced high green entrepreneurial intention. The core conditions of GEI1a, GEI1b, and GEI1c are the same, and the core conditions of GEI3a, GEI3b, and GEI3c are also the same, which constitute the second-order equivalent configuration (Fiss, 2011). Table 4 shows that the consistency indexes of the seven configurations are more than 0.850, thus indicating that the seven configurations are sufficient to explain the results of the high green entrepreneurial intention of college students. In addition, Table 4 shows that the overall coverage is 0.724507, which indicates that the seven configurations can explain 72% of college students’ high green entrepreneurial intention.

**TABLE 4:** Configurations sufficient for high green entrepreneurial intention.

	GEI1a	GEI1b	GEI1c	GEI2	GEI3a	GEI3b	GEI3c
Entrepreneurial creativity	•	•	•	•		⊗	⋅
Future self-continuity	⋅		⊗	⋅	•	•	•
Green cognition	•	•	•		⋅		
Entrepreneurship culture		⋅	⊗	⊗	•	•	•
Entrepreneurship education	⋅	⋅	⊗	⊗	•	•	•
Financial support			⊗	•		⊗	⋅
Consistency	0.872454	0.907147	0.968421	0.939394	0.881971	0.919973	0.896141
Raw coverage	0.416233	0.536951	0.0812641	0.136912	0.439297	0.132005	0.27353
Unique coverage	0.0213956	0.144077	0.0146236	0.0327805	0.0262048	0.0140347	0.0153106
Overall consistency	0.887793						
Overall coverage	0.724507						

*“⋅” or “•” indicates the existence of the variable; “⊗” or “⊗” indicates that the variable does not exist; “Blank” indicates that the condition may or may not exist in the combination; “•” or “⊗” denotes the core variable (the variables existing in both the intermediate solution and parsimonious solution are the core variables, which have an important impact on the results); “⋅” or “⊗” means the auxiliary variable (the variable with only intermediate solution is the auxiliary variable).*

GEI1a, GEI1b, and GEI1c are all related to high green entrepreneurial intention, and the three configurations show that entrepreneurial creativity and green cognition are the core conditions of the three configurations. However, the peripheral conditions of the three configurations were different. In the configuration of GEI1b, regardless of whether future self-continuity and financial support are sufficient or not, as long as entrepreneurial creativity and green cognition are high, college students will have a high green entrepreneurial intention. GEI1b has the largest raw coverage (54%) and unique coverage (14%), which is the main configuration leading to college students’ high green entrepreneurial intention. Comparing GEI1a and GEI1b, when entrepreneurship education exists, regardless of whether financial support is sufficient or not and whether future self-continuity and entrepreneurship culture are conducive to college students’ green entrepreneurial intention, as long as entrepreneurial creativity and green cognition are high, high green entrepreneurial intention can be generated among college students. Comparing GEI1c and GEI1b, regardless of whether there are adverse future self-continuity and financial support, and whether entrepreneurship culture and entrepreneurship education are conducive to college students’ green entrepreneurial intention, as long as entrepreneurial creativity and green cognition are high, college students will have a higher green entrepreneurial intention. By comparing these three configurations, it is evident that compared with the four conditions of future self-continuity, entrepreneurship culture, entrepreneurship education, and financial support, entrepreneurial creativity and green cognition play a crucial role in college students’ high green entrepreneurial intention. Enterprises with high entrepreneurial creativity have flexible green cognition and thinking, which further identifies the opportunities and potential benefits of green entrepreneurship. In addition, entrepreneurs with a high degree of green cognition can pay more attention to environmental issues than traditional entrepreneurs. They are aware of the possible impact of green entrepreneurial decisions on the environment; thus, they will creatively build cognition and put forward entrepreneurial thinking that is conducive to environmental issues (Jiang et al., 2020). Therefore, college students with high entrepreneurial creativity and green cognition will be more interested in green entrepreneurship. Therefore, these three conditions of high green entrepreneurial intention are called entrepreneurial creativity—green cognition—the dominant path.

The GEI2 configuration shows that regardless of whether green cognition exists or not, entrepreneurship culture and entrepreneurship education are poor, and if entrepreneurial creativity and financial support are high, high green entrepreneurial intention can be generated among college students. This result could be owing to fact that college students with high entrepreneurial creativity have more flexibility, exploratory mindset, and innovative thinking, can easily identify green entrepreneurship opportunities, and are more willing to participate in green entrepreneurship (Jiang et al., 2020). In addition, college students’ entrepreneurial intention is easily affected by the external economic environment (Yao et al., 2016)—the support of financial capital (Welter, 2011), such as start-up funds and low interest loans, can stimulate their entrepreneurial intention. Therefore, this group of conditions of high green entrepreneurial intention is called entrepreneurial creativity—financial support—the dominant path.

The configurations of GEI3a, GEI3b, and GEI3c show that their core conditions are consistent, that is, as long future self-continuity, entrepreneurship culture, and entrepreneurship education exist, high green entrepreneurial intention can be generated among college students. Among them, GEI3a has the largest raw coverage (44%) and unique coverage (2.6%), and it is the main configuration leading to college students’ high green entrepreneurial intention. The GEI3a configuration shows that regardless of whether entrepreneurial creativity and financial support exist or not, compared with green cognition, as long the future self-continuity, entrepreneurship culture, and entrepreneurship education level are high, college students will have high green entrepreneurial intention. The GEI3b configuration shows that when entrepreneurial creativity and financial support do not exist, and regardless of whether green cognition appears or not, as long the future self-continuity, entrepreneurship culture, and entrepreneurship education level are high, green entrepreneurial intention will be high among college students. The GEI3c configuration shows that regardless of whether there is green cognition or not, compared with entrepreneurial creativity and financial support, future self-continuity, entrepreneurship culture, and entrepreneurship education play a more important role in promoting college students’ green entrepreneurial intention. This result could be owing to the higher degree of future self-continuity, which indicates that the psychological connection between the future self and the present self is closer, and individuals may be able to further enhance entrepreneurial intention in the future (Bartels and Urminsky, 2011). Countries or regions with active entrepreneurial culture can promote individual entrepreneurship, and individuals can use available resources to develop entrepreneurship (Tiessen, 1997). Green entrepreneurship education differs from traditional business education. It emphasizes the orientation of entrepreneurial decision-making and entrepreneurial activities to the sustainable development of the environment to improve individual entrepreneurial inspiration, entrepreneurial knowledge, and entrepreneurial skills (Agu et al., 2021), which can effectively adjust individual entrepreneurial development direction and entrepreneurial mode (Kuckertz and Wagner, 2010). This fact implies that green entrepreneurship education can stimulate people to prepare for sustainable development. Therefore, those with good green entrepreneurship education can show higher green entrepreneurial intention. The green entrepreneurial intention of these three groups of college students under high conditions is future self-continuity—entrepreneurship culture—entrepreneurship education—dominant path.

### Sensitivity Analysis

Furthermore, a sensitivity analysis was performed to evaluate the robustness of the results in different ensemble calibration sets (Garcia-Castro and Francoeur, 2016). There are two types of robustness tests: the set theory specific robustness test and the statistical theory specific robustness test (Kim, 2013). The specific robustness testing methods of set theory mainly include adjusting the consistency threshold and calibration threshold, eliminating or supplementing cases, and changing the frequency of cases. The specific test methods of statistical theory mainly include changing the measurement methods and the data sources, increasing the reason conditions related to the results, and so forth. The two criteria used to judge the robustness of the QCA results are the set relation state and the difference of the fitting parameter. If the state of the set relation is a clear subset relation between different configurations (condition configurations) caused by different operations, the result is robust, and the difference in the fitting parameter implies that for divergent operations, the variation in the fitting parameter will not have a substantial impact on the interpretation of the results. Based on this fact, this study changed the consistency threshold from 0.8 to 0.85, and other conditions remained unchanged. The number of solutions, overall consistency, and overall coverage of the changed results were completely consistent with the original results, which showed that the explanation of the effect of condition configurations on college students’ green entrepreneurial intention had no substantial change, and therefore, it is robust.

## Discussion and Conclusion

Previous studies mainly focused on the impact of net effect of factors on green entrepreneurial intention among college students (Jiang et al., 2020; Soomro et al., 2020; Yi, 2020), and ignored the complex impact of multiple factors on the results. Therefore, this study applied configuration thinking to analyze the causal complex mechanism of college students’ high green entrepreneurial intention from two levels of entrepreneurs’ individual characteristics and institutional theory framework. The individual characteristics are entrepreneurial creativity and future self-continuity, and the institutional theory framework components are green cognition, entrepreneurship culture, entrepreneurship education, and financial support. Based on a questionnaire survey of 207 college students in China’s coastal areas, this study explored how six conditional factors affected college students’ green entrepreneurial intention. The results showed that the relationship between the condition and outcome factors is complex. There are seven configurations to generate college students’ high green entrepreneurial intention, which can be further divided into three main dominant paths. First, the entrepreneurial creativity—green cognition—dominant path is the interaction between high entrepreneurial creativity and a high green cognition level. Second, the entrepreneurial creativity—financial support—dominant path is the interaction between high entrepreneurial creativity and sufficient financial support. Third, the interaction between high future self-continuity, active entrepreneurship culture, and diversified entrepreneurship education led to future self-continuity—entrepreneurship culture—entrepreneurship education—dominant path. Among the seven configurations, entrepreneurial creativity appeared in four configurations, which indicates that this condition variable has a more profound influence on the high green entrepreneurial intention of college students than any other condition variable. This result may be because green entrepreneurship is different from ordinary entrepreneurship as it gives high importance to the concept of environmental protection; thus, green entrepreneurship is not profit oriented. Individuals with active entrepreneurial creativity think more innovatively and can find internal resources and breakthrough points more flexibly. Mere active entrepreneurial creativity cannot help achieve high green entrepreneurial intention; it also requires the combination of other condition variables. In addition, financial support appeared only in one of the seven configurations that affect high green entrepreneurial intention. This result shows that although financial support is a key factor in the growth of green start-ups (Bergset, 2018; Demirel et al., 2019), it is not the most important aspect that influences their intention to engage in green entrepreneurship. This result could be owing to fact that college students have not actually participated in actual entrepreneurship and have no practical experience in handling financial difficulties. Rather, they give importance to the opportunities and idea of green entrepreneurship, and how they can start a venture.

The practical implications of this study lie in the following three aspects. From the perspective of the entrepreneurial creativity—green cognition—dominant path, regardless of whether there is future self-continuity, entrepreneurship culture, entrepreneurship education, and financial support or not, the colleges can provide diversified creative thinking courses, focus on the cultivation of college students’ entrepreneurial creativity, and actively guide college students’ green awareness and sustainable development knowledge to encourage college students’ green entrepreneurial intention. Second, from the perspective of an entrepreneurial creativity—financial support—dominant path, in a case where there are no green entrepreneurship education courses in colleges and the country or region do not support green entrepreneurship culture, if the colleges want to improve the green entrepreneurial intention of college students, the entrepreneurship teachers have to guide the students to ameliorate their entrepreneurial creativity. Additionally, the state and colleges need to provide diversified financial support, such as entrepreneurial start-up funds, entrepreneurial subsidies, and low interest loans, to broaden financing channels and extend the financing environment to activate the green entrepreneurial intention of college students. Third, from the perspective of the future self-continuity—entrepreneurship culture—entrepreneurship education—dominant path, in the case of poor entrepreneurial creativity, green cognition, and financial support, it is necessary to improve the individual’s future self-continuity. Countries or regions need to further promote green entrepreneurial culture. Additionally, colleges should provide green entrepreneurship education to promote college students’ green entrepreneurial willingness.

The limitation of this study is that it collected data only from college students in the coastal areas of China. In the future, follow-up research can be extended globally, expanding the sample range of college students’ green entrepreneurial intention to improve the applicability of the conclusions. The factors that affect the green entrepreneurial intention of college students can be considered from the perspective of personality. This study created a framework using entrepreneurs’ individual characteristics and institutional theory framework, and future research can explore this topic further from the perspective of personality traits. Moreover, in-depth interviews can be used to revise and supplement the results of this study. In the future, we can collected data for in-depth analysis of specific phenomena as follows: (1) How do you understand green entrepreneurship? (2) What other factors do you think affect green entrepreneurship? (3) What factors do you think play the most important role in promoting green entrepreneurship? (4) Does colleges actively encourage college students to carry out green entrepreneurship? (5) What are advantages that college students have in green entrepreneurship? (6)What are the disadvantages? (7) Which factor has a greater impact on your green entrepreneurship activities? (8) Have your classmates or relatives and friends carried out green entrepreneurship? (9) How much do you think they affect you? and (10) Do you think financial support does not play a significant role in college students’ green entrepreneurial intention? Therefore, future research can consider in-depth interviews and tracking to better analyze why college students participate in green entrepreneurship.

## Data Availability Statement

The raw data supporting the conclusions of this article will be made available by the authors, without undue reservation.

## Author Contributions

YZ collected and analyzed the data. YZ and XC wrote the manuscript. SH, XC, and YZ conceived and designed the study and contributed to the article and approved the article can be published.

## Conflict of Interest

The authors declare that the research was conducted in the absence of any commercial or financial relationships that could be construed as a potential conflict of interest.

## Publisher’s Note

All claims expressed in this article are solely those of the authors and do not necessarily represent those of their affiliated organizations, or those of the publisher, the editors and the reviewers. Any product that may be evaluated in this article, or claim that may be made by its manufacturer, is not guaranteed or endorsed by the publisher.
